# Synergistic Ecoclimate Teleconnections from Forest Loss in Different Regions Structure Global Ecological Responses

**DOI:** 10.1371/journal.pone.0165042

**Published:** 2016-11-16

**Authors:** Elizabeth S. Garcia, Abigail L. S. Swann, Juan C. Villegas, David D. Breshears, Darin J. Law, Scott R. Saleska, Scott C. Stark

**Affiliations:** 1 Department of Atmospheric Sciences, University of Washington, Seattle, WA, 98195, United States of America; 2 Department of Biology, University of Washington, Seattle, WA, 98195, United States of America; 3 Escuela Ambiental, Universidad de Antioquia, calle 67 No. 53–108 Medellín, Colombia; 4 School of Natural Resources and the Environment, University of Arizona, 1064 E. Lowell St, ENRII, N227, Tucson, AZ, 85721, United States of America; 5 Department of Ecology and Evolutionary Biology, University of Arizona, Tucson, AZ, 85721, United States of America; 6 Department of Forestry, Michigan State University, East Lansing, MI, 48824, United States of America; Kerala Forest Research Institute, INDIA

## Abstract

Forest loss in hotspots around the world impacts not only local climate where loss occurs, but also influences climate and vegetation in remote parts of the globe through ecoclimate teleconnections. The magnitude and mechanism of remote impacts likely depends on the location and distribution of forest loss hotspots, but the nature of these dependencies has not been investigated. We use global climate model simulations to estimate the distribution of ecologically-relevant climate changes resulting from forest loss in two hotspot regions: western North America (wNA), which is experiencing accelerated dieoff, and the Amazon basin, which is subject to high rates of deforestation. The remote climatic and ecological net effects of simultaneous forest loss in both regions differed from the combined effects of loss from the two regions simulated separately, as evident in three impacted areas. Eastern South American Gross Primary Productivity (GPP) increased due to changes in seasonal rainfall associated with Amazon forest loss and changes in temperature related to wNA forest loss. Eurasia’s GPP declined with wNA forest loss due to cooling temperatures increasing soil ice volume. Southeastern North American productivity increased with simultaneous forest loss, but declined with only wNA forest loss due to changes in VPD. Our results illustrate the need for a new generation of local-to-global scale analyses to identify potential ecoclimate teleconnections, their underlying mechanisms, and most importantly, their synergistic interactions, to predict the responses to increasing forest loss under future land use change and climate change.

## Introduction

Forest loss is progressing globally from deforestation associated with land use change [1,2] and tree mortality resulting from climate change [3,4]. Large portions of forest have been lost to deforestation, particularly in the tropics, where the highest rates of conversion from native forest into agricultural and other productive uses have occurred in the 20th century [1,2], further exacerbated by increasing fire activity [5]. Concurrently, extensive tree mortality related to drought, heat and associated pests and pathogens is increasing globally [3,4], further contributing to global forest loss. Such mortality events have been particularly pronounced in western North America [6–8]. Tree mortality in the Amazon has also increased recently in response to intense drought [9,10]. Such broad-scale tree loss events have important implications for land surface—atmosphere interactions.

**Fig 1:**
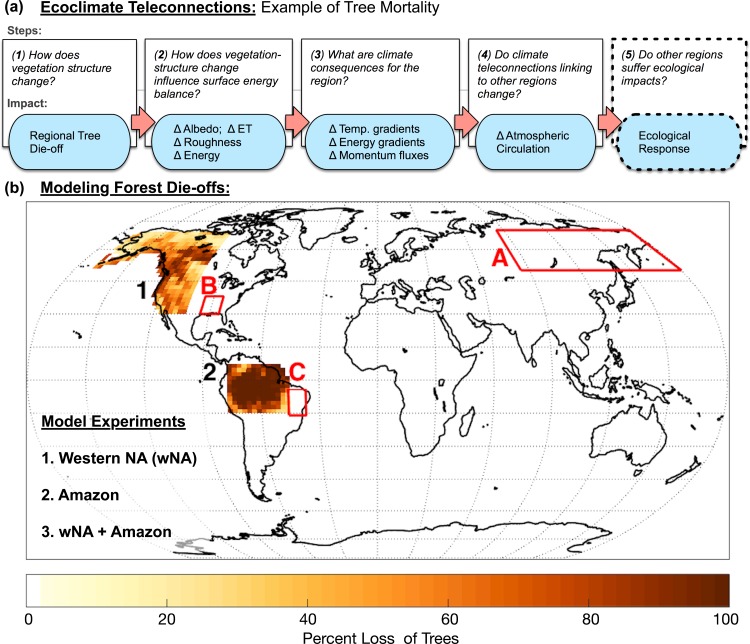
Ecoclimate teleconnection concepts and simulation experiments. (a) Ecoclimate teleconnections propagate land-atmosphere energy disturbances from the local region of disturbance to remote regions potentially having ecological consequences (taken from [14]). (b) In our experiments, forest is converted to grass in three scenarios to illustrate the ecoclimate teleconnections: (1) western North America (wNA) only; (2) the Amazon basin only; and (3) both wNA and the Amazon simultaneously (wNA+Amazon). Red boxes highlight three focal areas that show changes in GPP associated with teleconnections with mechanistic relationships discuss in the text: (A) Eurasia; (B) southeastern North America (SENA); and (C) eastern South America (ESA).

Broad-scale changes in vegetation in general, and tree loss in particular, have pronounced effects on climate processes through biogeophysical mechanisms such as albedo, evapotranspiration (ET), and carbon dioxide exchange with the atmosphere [11]. The resultant local changes in microclimate can have broader-scale impacts on climate and vegetation elsewhere via “ecoclimate teleconnections” [12,13]. Assessing ecoclimate teleconnections requires explicit consideration of vegetation-climate feedbacks through five steps ([14]; Fig 1A): “(1) How does the land surface change in terms of vegetation structure?; (2) How does the vegetation-structure change influence albedo and other components of land surface energy balance?; (3) What are the consequences of the changes in vegetation structure and energy balance for the region?; (4) What climate teleconnections link the initially impacted region to other regions?; and (5) What type of secondary ecological impacts does this produce in another teleconnected region of interest?"

Modeling studies have identified potential climate teleconnections associated with broad-scale tree loss. Forests can affect the global climate system by altering large-scale patterns in atmospheric waves and jet streams, a mechanism termed “teleconnection patterns” (e.g., [12, 15–19]). Climate teleconnections occur due to atmospheric mechanisms by which local energy imbalances due to changes in the land surface properties are propagated remotely. How a forest disturbance impacts climate strongly depends on forest type and its latitudinal location [20]. For example, removing dark boreal forests primarily leads to global cooling through the radiative effects of increasing local albedo [21–23]. Tropical forest removal leads to local warming due to reduction in latent heat fluxes [24] and also alters hydrometeorology in mid- and northern latitudes [15,25]. Mid-latitude forest loss or gain can shift tropical precipitation bands [12,18,26]. Most of the relevant research to date has focused on the first four steps of ecoclimate teleconnection assessment (Fig 1A), with only a few also considering resultant ecological effects on vegetation in the remote, teleconnected location [12,14].

To date, studies have considered tree loss events from a single regional location, most often associated with a latitudinal band [20,24]. Tree loss, however, will likely occur more regionally, such as the ongoing wNA tree die-off or ongoing deforestation of the Amazon. Because any given tree loss event has the potential to affect ecoclimate teleconnections, and tree loss is occurring simultaneously in disparate regions, assessments are needed that account for concurrent tree loss events to assess whether ecological responses differ from those of individual loss events. Ecological effects can vary among remote teleconnected locations [14] and can be highly sensitive to changing climate regimes [27], although mechanisms associated with given ecological responses have not generally been explicitly evaluated. To address future challenges related to climate change and land use, it will be important to assess the local impacts of tree loss on climate, climatic and ecological consequences, as well as the mechanisms that drive ecological responses in remote teleconnected areas. In short, the extent and rapidity of ongoing and projected tree loss necessitates consideration of how tree loss in disparate regions might differ and potentially interact, as well as identifying the mechanisms associated with predicted responses.

To address this issue, we investigate the climatic consequences and resulting ecological impacts of forest conversion to grass in two large regions individually: (1) western North America (wNA), and (2) the Amazon basin, and (3) the combination of wNA and the Amazon (wNA+Amazon) (Fig 1B). These experimental regions are intentionally extreme in vegetation consequences (i.e., full conversion from forest to grassland functional types without ecologically intermediate types in order to act as bounding calculations that we use to reveal remote changes in GPP. Based on the results from these experiments, we highlight significant ecologically-relevant climate impacts and the mechanisms that drive ecological change in three impacted regions: Eurasia, southeastern North America and eastern South America. We discuss how the climatic and ecological effect of forest loss combined (wNA+Amazon) from both loss regions did or did not differ from the effect of loss from the regions individually in the three impacted areas. More generally, this work provides an example of how forest change and climate interact to impact remote ecosystems.

## Methods

### Model

We use the National Center for Atmospheric Research (NCAR) Community Earth System Model version 1.3 (CESM) that couples the Community Atmosphere Model version 5 (CAM5) [28] to the Community Land Model (CLM4.5; [29]), the CICE4 sea ice model [30], and implements a slab ocean with prescribed heat transport derived from a fully-coupled ocean-atmosphere simulation [28]. The slab ocean model is a computationally efficient scheme that allows sea surface temperatures to interact with the atmosphere; and is necessary for propagating energy imbalances due to land cover change that lead to shifts in precipitation. Biogeophysical interactions between plants and the atmosphere include shortwave and longwave radiation interaction with the plant canopy, stomatal resistance, and wind turbulence. Stomatal resistance is calculated using a Ball-Berry formation [29,31] and is a function of leaf photosynthesis, relative humidity, atmospheric pressure, and plant functional type parameters that result in a maximum stomatal resistance of 20000 s/m. Leaf area and biomass respond to climate which can lead to changes in albedo and transpiration. The land model has an interactive carbon cycle with the default nitrogen cycle modified to be constant, following [32]. Model simulations are conducted at a resolution of 1.9° latitude by 2.5° longitude and are run for 100 years. Climate and terrestrial variables (LAI, temperature, precipitation) reach equilibrium after approximately 20 years of model spin up. The spin up period is discarded and results analyze time series from the remaining 80 years. All runs are conducted using preindustrial (1850) land use conditions in order to conform with our best-available slab ocean model data; however the orbital conditions are set for the year 2000. This study focuses only on biogeophysical effects of forest disturbance, so atmospheric CO_2_ concentrations are held fixed to present day conditions of 367 ppm.

### Modeling experiments

Our experiments replace forests with C3 grasses in three scenarios (Fig 1B): (i) in the western half of North America (wNA) (190 to 255°W and 30 to 70°N); (ii) the Amazon basin (-16°S to 8°N and 282 to 313°W); and (iii) both wNA and the Amazon (wNA+Amazon). Forest cover is defined as the historical land cover type default provided by CLM. The distribution of plant functional types is fixed throughout the experiments.

Results are analyzed by comparing experiments with control simulations. We report results for the difference between experiment (wNA, Amazon, wNA+Amazon) and the control run. We identify mechanisms associated with ecological responses (i.e., GPP) in a given location by reviewing associated variables (e.g., temperature, precipitation, VPD, soil moisture). We also compare the sum of two experiments (wNA and Amazon) to the simulation representing simultaneous deforestation (wNA+Amazon) in order to explore non-combinatorial amplifications or offsets introduced by simultaneous vegetation disturbance and associated teleconnected climate alterations. Statistical significance between the experiments and the control is tested using a student’s *t*-test and significance is determined using resultant *p* values, where a *p* value less than or equal to 0.05 allows rejecting the null hypothesis that the differences should be zero. We use a lagged auto-correlation of 2 years or less for all variables and 40 degrees of freedom for the 80 years of computed results. We analyze spatial patterns of precipitation globally associated with forest loss by calculating shifts in the global tropical precipitation band, the Inter-Tropical Convergence Zone (ITCZ), associated with changes in cross-equatorial atmospheric heat transport using equation 2.21 from [33]. Previous modeling work has shown a correlation between cross-equatorial energy transport and the location of tropical rain bands associated with the Hadley cell [34–36]. A north- or southward shift in the Hadley cell facilitates heat transport when forest loss in either hemisphere results in an energetic imbalance between hemispheres. This Hadley circulation change results in a shift in the tropical precipitation band, in agreement with the dynamics of prior model studies that have identified interhemisperical ecoclimate teleconnections [12].

## Results

### Global climate and ecological response to regional vegetation change

**Fig 2:**
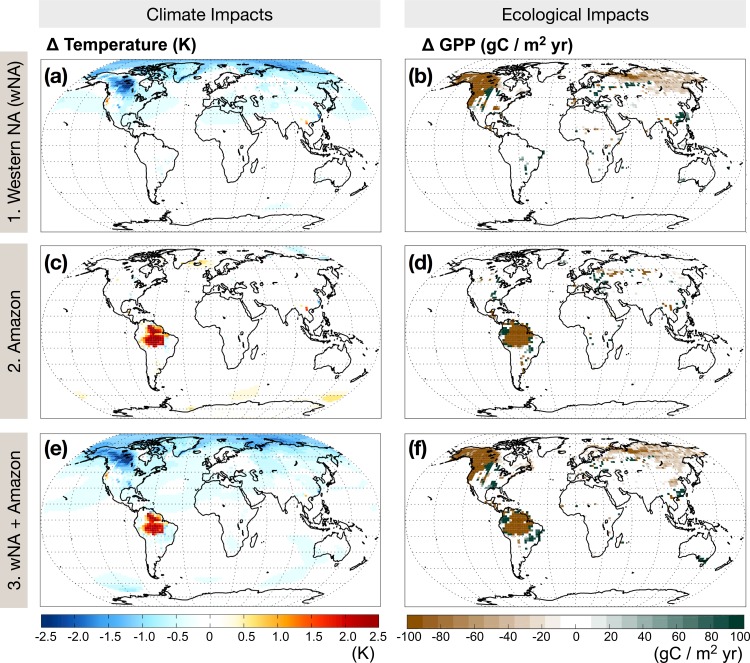
Climatic and ecological responses to Amazon and wNA forest loss. Anomalies in annually averaged temperature in Kelvin (panels a, c, e) and annual gross primary productivity in gC/m^2^yr (panels b, d, f), calculated as the difference between the control and experimental case of forest loss in western North America alone (wNA, panels a, b); the Amazon alone (panels c, d) and the wNA+Amazon together (panels e, f). Values that do not pass a significance test at 95% confidence are not included.

Model results show that regional forest loss in all experiments leads to global cooling (Fig 2A, 2C and 2D) with the cooling dominated by wNA forest loss. Loss of wNA forests leads to global average cooling of -0.2 K; cooling occurs primarily in the northern latitudes with the exception of local warming within the disturbance area (Fig 2A). Loss of Amazon forests leads to local increases in average temperatures due to decreases in evaporative cooling, however there is generally little change in global average temperature (Fig 2C). The combination wNA+Amazon experiment reflects the northern hemispheric cooling and Amazon basin warming that is present in the individual experiments. However, the magnitude and spatial pattern of cooling in the northern latitudes is not an additive combination of the individual experiments (0.04 K warmer than the combination experiment). This suggests that Amazon forest loss offsets the cooling effect imposed by wNA forest loss and is evidence of a nonlinear geographic response due to forest loss in the two locations.

Regional forest loss also modifies global precipitation patterns, though the majority of the spatial pattern and magnitude of change in this highly time-variable quantity does not pass a significance test (S1 Fig). Our results generally suggest that wNA forest loss shifts the ITCZ southward, with corresponding decreases in precipitation throughout the northern tropics (0-10N). There is an opposite shift in ITCZ location with Amazon forest loss: the ITCZ band shifts slightly northward leading to increases in precipitation over Asia’s southeastern tropics, and declines in NA’s Pacific Northwest. The combined wNA+Amazon results show a stronger southern shift the ITCZ band than the wNA case.

Shifts in global temperature and precipitation patterns contribute to significant responses estimated in terrestrial GPP in remote regions (Fig 2). We next describe results in regions remote to the imposed disturbance scenarios (highlighted in Fig 1B): Eurasia, southeastern North America (SENA), and eastern South America (ESA). We focus on these regions for several reasons. First, they are the largest regions that demonstrate significant changes in GPP. Second, we are able to identify climate mechanisms that directly contribute to the changes in GPP. Third, each region illustrates a unique type of response—i.e., strength in GPP response relative to proximity to disturbance, variable climate response with unique forest loss, and nonlinear GPP response with individual versus combined forest disturbances

### Remote forest response to change in vegetation—regional examples

#### Eurasia

**Fig 3:**
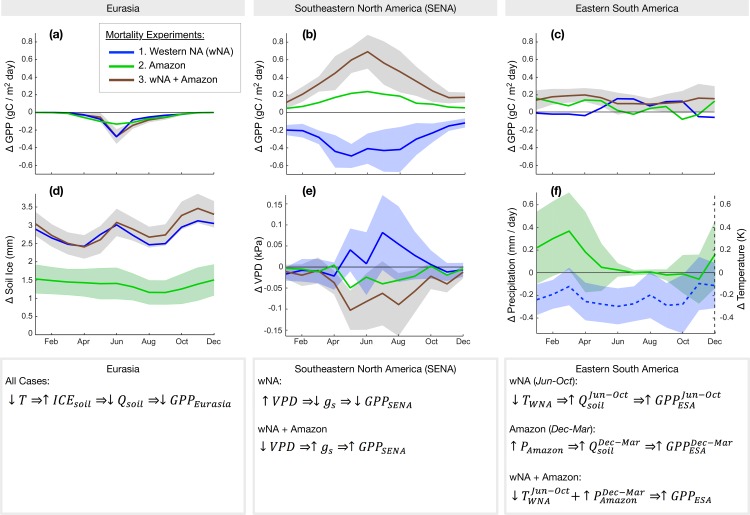
Mechanisms driving ecological responses. Anomalies in averaged monthly GPP in gC/m^2^/day between the control and experimental scenarios in three regions (see Fig 1B): (a) Eurasia, (b) southeastern North America (SENA), and (c) eastern South America (ESA). Key climatically-influenced mechanisms contributing to changes in GPP include: (d) conversion of soil moisture to ice in Eurasia; (e) VPD-induced responses in stomatal conductance (g_s_) in SENA; (f) Amazon forest loss alone leads to increases in precipitation in Dec-Mar and wNA forest loss alone leads to declines in temperatures in Jun-Oct. These seasonal changes contribute to an annual increase in GPP with simultaneous wNA+Amazon forest loss due to release from soil moisture limitation (not shown). Shading in *a*–*e* shows the ±1 SE estimated from the control.

A large decline in GPP occurs across most of Eurasia during the growing season in all three experiments. The greatest decline in Eurasia’s GPP occurs with the two experiments including forest loss in wNA (Fig 3A). GPP is also significantly reduced in the Amazon experiment relative to the control scenario, however its peak decline in June (-0.12 gC/m^2^/day) is approximately half of the peak decline calculated in the scenarios with loss of wNA forests (0.25 gC/m^2^/day, Fig 3A). Surface temperatures are cooler in this region in all three experiments and the wNA and the wNA+Amazon experiments are significantly cooler relative to the control (S1 Table). The cooler surface temperatures convert liquid soil moisture to significant increases in soil ice (Fig 3D). Without a corresponding change (i.e., increase) in precipitation, this leads to a reduction of plant available soil moisture which increases plant water stress through the growing season, thereby reducing GPP.

#### Southeastern North America (SENA)

Southeastern North America (SENA) experiences significant declines in GPP throughout the year with forest loss in wNA (Fig 3B). In contrast, forest loss in wNA+Amazon leads to significant increases in SENA’s GPP throughout most of the year (Fig 3B). Increases in SENA’s GPP with Amazon forest loss and the climate mechanisms responsible for these changes are similar to the result of the combined experiment, however they do not pass our significance threshold, so we focus on climate mechanisms related to wNA and wNA+Amazon results only.

The diverging responses in SENA’s GPP with wNA versus wNA+Amazon forest loss correspond to changes in regional vapor pressure deficit (VPD, Fig 3E). For wNA forest loss, VPD increases during the growing season (Apr-Aug). An increase in VPD decreases plant stomatal conductance, leading to declines in carbon assimilation and GPP. Conversely, forest loss over wNA+Amazon drives declines in VPD during the growing season in SENA (Fig 3E), which increases stomatal conductance, and GPP (Fig 3B). VPD, calculated as e_st_(1-RH), is changed with both relative humidity (RH), a moisture index, and changes in the saturated vapor pressure (e_st_), a temperature index. With wNA forest loss, there are significant declines in both precipitation and temperature during the early growing season, however it is the change in the relative humidity that dominates the observed increase in VPD. Similarly, the decline in temperature rather than relative humidity dominates the decline in VPD with forest loss in wNA+Amazon; declines in temperature throughout the year, in combination with only modest declines in precipitation, lead to VPD reductions. The GPP response in SENA to remote regional forest loss demonstrates that ecological responses may differ depending on the location of forest loss and associated mechanisms of climate teleconnections.

#### Eastern South America

Eastern South America’s GPP increases throughout the entire year with wNA+Amazon forest loss (Fig 3C). We note that this region is external to the area where forest loss was imposed in the Amazon. This annual increase is due to the combined, seasonally asynchronous, contributions of both regions to GPP increases (wNA: GPP increases May-Oct; Amazon: GPP increases Dec-May; Fig 3C), which are driven by different mechanisms. Temperature declines in this region throughout the year with wNA forest loss result in a significant release from soil moisture limitation for productivity and transpiration from May-Oct. Amazon forest loss drives significant increases in precipitation from Dec-May (April excluded, Fig 3F), which corresponds temporally to increased soil moisture saturation and decreases in later, growing season limitations. Cooling during the period of seasonal water limitation reduces modeled plant water stress. Our results show that the loss of each individual forest for the wNA+Amazon scenario contributes to complementing seasonal reductions in water stress that result in year-round increases in GPP. This is an example of a temporally linear combination of seasonal GPP impacts with loss of a tropical and temperate forest—i.e., an additive teleconnection impact.

## Discussion

Our results build on previous modeling efforts and show that regional scale forest loss impacts global climate circulation patterns, and extends the implications of these climate impacts to consequences for remote ecosystems. Additionally, our results also address the need to consider concurrent tree loss in disparate regions. Our results link disparate ecosystems through ecoclimate teleconnections, whereby disturbance in one location leads to an ecosystem response in a remote location. Pieces of this coupling have been introduced previously: that forest loss/gains perturb the surface energy budget sufficiently to influence global climate [15,25]; and that remote, large-scale climate patterns, such as El Nino Southern Oscillation (ENSO), impact ecosystem productivity [37,38]. However identifying ecoclimate teleconnections that link terrestrial disturbance to global climate patterns with explicit consideration of impacts to remote ecosystems has rarely been considered (excepting [12], [13]).

Our modeled changes in climate broadly agree with previous research showing that forest loss in western North America leads to global cooling and forest loss in the Amazon leads to local warming [20,21,39]. Though previous research has demonstrated the existence of climate teleconnections originating from forest disturbance [15,24,25], only limited work has shown the ecological impacts of these climate teleconnections [12,14] and none, to our knowledge, has explicitly considered the competing ecological impacts of disparate forest biomes. We also demonstrate that forest loss in western North American may lead to cooling in a similarly northern latitudinal band in Eurasia, suggesting that changes in Northern Hemisphere climate teleconnections [40] may have ecological impacts. This ecoclimate teleconnection would not have been apparent had we implemented a boreal forest loss across all of the northern latitudes (as in [24]). The ecological effect of forest loss in individual regions versus combined may include the possibilities of offsets and amplifications of the modeled effects, depending on where impacts are studied and what ecological mechanisms are implicated. We focus on comparing impacts from two particular forests that historically demonstrate vulnerability to increased rates of tree loss from dieback and/or deforestation.

The ecological impacts of climate teleconnections depend both on the origin of disturbance and the region of interest for assessing impacts. The ecosystem effects in the three regions described in this work demonstrate that forest loss in wNA and/or the Amazon may have synergistic ecological impacts. The critical climate impacts of teleconnections, and the local-scale ecological mechanisms implicated in modeled vegetation responses to teleconnections, differed across each example region. These results illustrate the first-order implications for global ecosystems resulting from regional forest disturbances. Our example experimental regions are intentionally extreme in both size and in vegetation consequences (i.e., full conversion from forest to grassland functional types without ecologically intermediate types or recovery is unlikely), thus acting as bounding calculations [14,24] that we use here to reveal specific mechanisms related to remote reductions in GPP. We note that we focus only on biogeophysical impacts, whereas biogeochemical impacts of CO2 associated with forest dieback could have distinct, large climatic effects [41]. However, recent work confirms that historical forest loss results in measurable climate impacts [42]. Future work will introduce disturbance scenarios that are based on observed forest loss (i.e., [43]). Our simulated GPP reductions, if sufficient in magnitude and duration, could result in tree mortality in the teleconnected impacted areas. Future coupling of demography with existing global land model predictions could enable assessment of these potentially important die-off responses [44], as well as implementation of more realistic reductions in tree loss to drive scenarios (i.e., enabling assessments of ecological changes less drastic or occurring on shorter time-scales than conversion from forest to grassland biomes).

Knowledge of these particular climate mechanisms can be used to distinguish subtle, but regionally important, vegetation effects amongst different types of climate disturbances arising from teleconnections. For example, mechanisms evaluated in this work revealed a cooling trend in Eurasia due to forest loss in western North America; this finding, however, contrasts with evidence of a warming trend in the Northern Hemisphere high latitudes with increased CO_2_ concentrations [45] that result in shifts in boreal forest range and disturbance regime [46]. Placing these ecoclimate consequences in a regional context also makes it possible to more directly compare local impacts with field measurements of vegetation change. New field techniques that facilitate measuring recent regional disturbances [14] are a necessary first step in quantifying land cover changes and energy balance response.

Our results illustrate the utility of global scale analyses that can aide in identifying potential ecoclimate teleconnections affected by vegetation change at regional scales, while simultaneously providing insight into the mechanisms of the associated ecological responses in affected regions. Ecoclimate teleconnections, if sufficiently strong, may need to be accounted for explicitly as carbon management systems become more globally coordinated (i.e., in response to the 21st Conference of the Parties United Nations [47]). Additionally, projections of large-scale forest die-off [48] and continued deforestation [2,43] further necessitate the explicit consideration of alterations to ecoclimate teleconnections. Our findings highlight how land surfaces are dynamically connected via different climate teleconnections and local ecological mechanisms, and that these linkages need to be further identified and factored into ecosystem assessment and management. In summary, these results demonstrate the potential for synergies and sensitivities of ecological response to forest loss in disparate regions via ecoclimate teleconnections, which will need to be accounted for as global forest loss increases and climate dynamics are altered in response to land use and climate change.

## Supporting Information

S1 Fig Anomalies in annual averaged precipitation. Anomalies in annual average precipitation in mm per day in three experiments: (a) wNA, (b) Amazon, (c) wNA+Amazon. Results are not masked for significance. Anomalies are calculated as the difference between the control and experimental cases. We include all values, including those that do not pass a significance test. Stippling indicates values that pass a significance test at the 95% threshold.
(PDF)

S1 Table Change in climate variables calculated as the area-weighted difference between the experimental and control case. Significance of change is indicated with p-values, reported in parentheses.
(DOCX)
