# Supplementary figures and images for: Synergistic Ecoclimate Teleconnections from Forest Loss in Different Regions Structure Global Ecological Responses
Source: PLoS One. 2016 Nov 16;11(11):e0165042. doi: 10.1371/journal.pone.0165042 (PMC5112850; doi:10.1371/journal.pone.0165042)

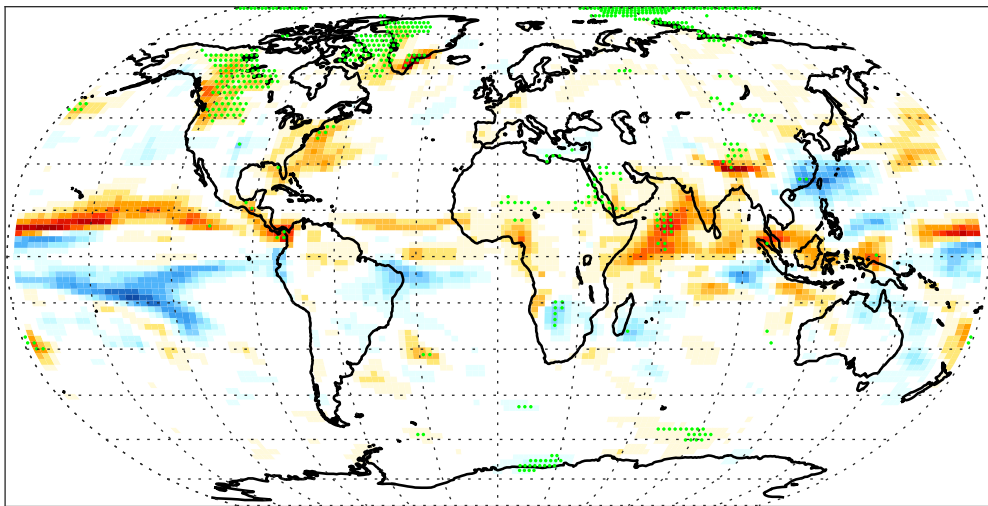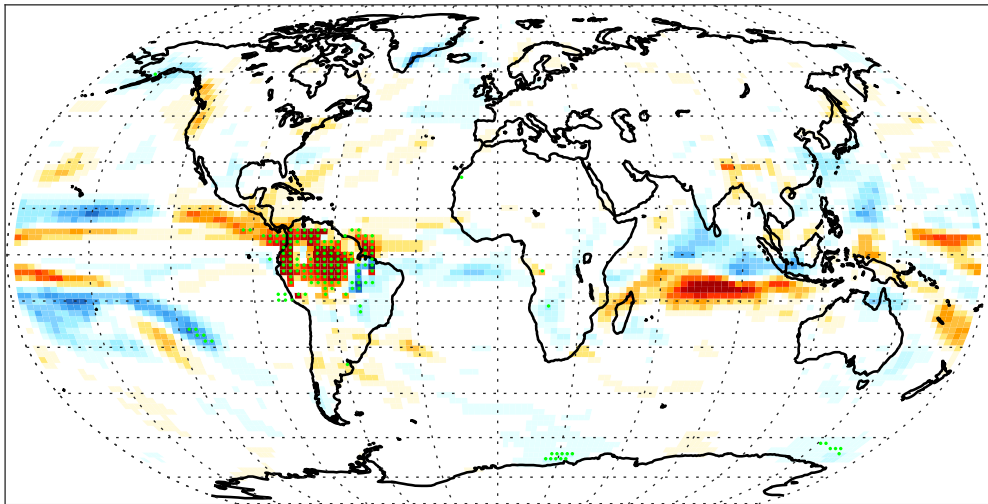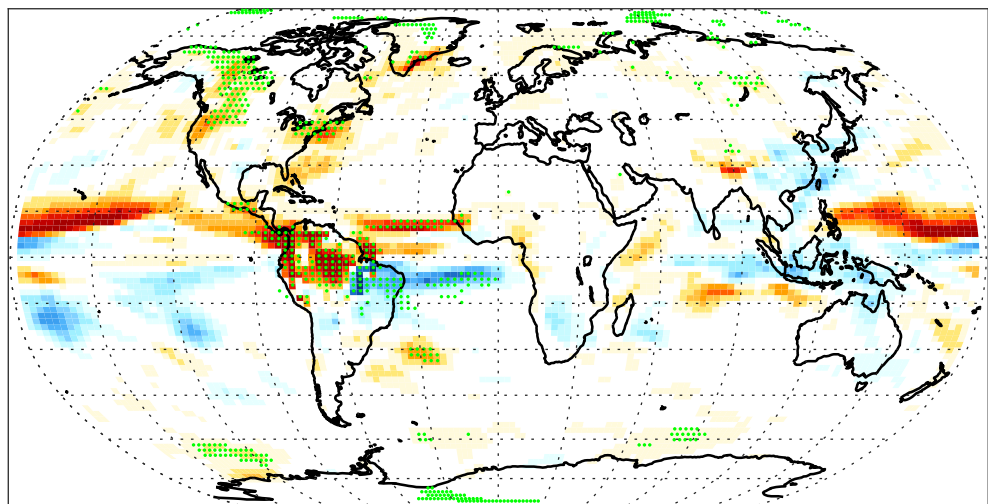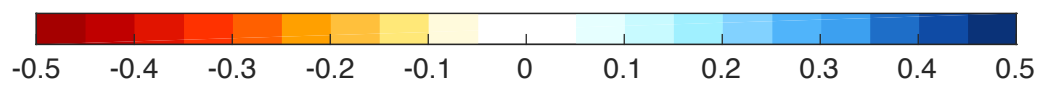

mm/day

Supplement: S1 Fig — Anomalies in annual average precipitation in mm per day in three experiments: (a) wNA, (b) Amazon, (c) wNA+Amazon. Results are not masked for significance. Anomalies are calculated as the difference between the control and experimental cases. We include all values, including those that do not pass a significance test. Stippling indicates values that pass a significance test at the 95% threshold. (PDF) [file pone.0165042.s001.pdf]
