# Supplementary material for: Synergistic Ecoclimate Teleconnections from Forest Loss in Different Regions Structure Global Ecological Responses
Source: PLoS One. 2016 Nov 16;11(11):e0165042. doi: 10.1371/journal.pone.0165042 (PMC5112850; doi:10.1371/journal.pone.0165042)
Supplement: S1 Table — Significance of change is indicated with p-values, reported in parentheses. (DOCX) [file pone.0165042.s002.docx]

**Supplemental Table 1.** Change in climate variables calculated as the area-weighted difference between the experimental and control case. Significance of change is indicated with p-values, reported in parentheses.

|  | ***Region*** | ***wNA*** | ***Amazon*** | ***wNA+Amazon*** |
| --- | --- | --- | --- | --- |
| ΔT (K/day) | Eurasia | *-*0.5 *(<0.001)* | -0.12 *(0.2)* | -0.7 *(<0.001)* |
| ΔVPD (kPa/day) | Southeastern North America | 1.9 *(0.1)* | -0.8 *(0.5)* | -0.81 *(0.3)* |
